# Estimation of Entropy for Inverse Lomax Distribution under Multiple Censored Data

**DOI:** 10.3390/e22060601

**Published:** 2020-05-28

**Authors:** Rashad A. R. Bantan, Mohammed Elgarhy, Christophe Chesneau, Farrukh Jamal

**Affiliations:** 1 Department of Marine Geology, Faculty of Marine Sience, King AbdulAziz University, Jeddah 21551, Saudi Arabia; rbantan@kau.edu.sa; 2 Valley High Institute for Management Finance and Information Systems, Obour, Qaliubia 11828, Egypt; m_elgarhy85@sva.edu.eg; 3 Department of Mathematics, Université de Caen, LMNO, Campus II, Science 3, 14032 Caen, France; 4 Department of Statistics, Govt. S.A Postgraduate College Dera Nawab Sahib, Bahawalpur, Punjab 63100, Pakistan; drfarrukh1982@gmail.com

**Keywords:** inverse Lomax distribution, Rényi entropy, *q*-entropy, multiple censored, simulation, 60E05, 62E15, 62F10

## Abstract

The inverse Lomax distribution has been widely used in many applied fields such as reliability, geophysics, economics and engineering sciences. In this paper, an unexplored practical problem involving the inverse Lomax distribution is investigated: the estimation of its entropy when multiple censored data are observed. To reach this goal, the entropy is defined through the Rényi and *q*-entropies, and we estimate them by combining the maximum likelihood and plugin methods. Then, numerical results are provided to show the behavior of the estimates at various sample sizes, with the determination of the mean squared errors, two-sided approximate confidence intervals and the corresponding average lengths. Our numerical investigations show that, when the sample size increases, the values of the mean squared errors and average lengths decrease. Also, when the censoring level decreases, the considered of Rényi and *q*-entropies estimates approach the true value. The obtained results validate the usefulness and efficiency of the method. An application to two real life data sets is given.

## 1. Introduction

Entropy is one of the most popular measure of uncertainty. As former mathematical work, Reference [1] proposed a theory on the concept of entropy, with numerical indicators as well. This theory was enhanced by numerous other entropy-like measures, arising from various applied fields. In this regard, a complete survey can be found in Reference [2]. Here, we focus our attention on two of the most famous entropy measures—the Rényi entopy by Reference [3] and the *q*-entropy by Reference [4] (also called Tsallis entropy). The Rényi entropy finds its source in the information theory and the *q*-entropy comes from the statistical physics, with a plethora of applications in their respective fields. For a random variable *X* having the probability density function (pdf) f(x;φ), where φ represents the corresponding parameters, these two entropy measures are, respectively, defined by

(1) $$\begin{array}{c} I_{\delta }\left(X;\varphi \right)=\frac{1}{1-\delta }\log\left[\int_{-\infty }^{+\infty }f{\left(x;\varphi \right)}^{\delta }dx\right], \end{array}$$

where δ≠1 and δ>0, and

(2) $$\begin{array}{c} H_{q}\left(X;\varphi \right)=\frac{1}{q-1}\left[1-\int_{-\infty }^{+\infty }f{\left(x;\varphi \right)}^{q}dx\right], \end{array}$$

where q≠1 and q>0. In particular, the Rényi entropy contains several well-known entropy measures, such as the Hartley entropy given by $\lim_{\delta \to 0}I_{\delta }\left(X;\varphi \right)$, the Shannon entropy obtained as $\lim_{\delta \to 1}I_{\delta }\left(X;\varphi \right)$ and the collision entropy given as $I_{2}\left(X;\varphi \right)$.

In most of the observed phenomena, at least φ is unknown, and the entropy as well. For this reason, the theoretical or practical statistical treatment of the entropy have been the object of all the attentions, in various settings. Among the notable studies in this regard, we may refer the reader to Reference [5] discussing the entropy of ordered sequences and order statistic, Reference [6] focusing on the entropy of upper record values, Reference [7] proposing the entropy of hybrid censoring schemes, Reference [8] discussing the entropy of progressively censored samples, Reference [9] investigating the estimation of the entropy for the Weibull distribution under the progressive censoring scheme, Reference [10] using the maximum likelihood and Bayes estimators via doubly-generalized Type II hybrid censored samples to estimate the entropy for the Rayleigh distribution, Reference [11] studying the Bayes estimation of the entropy for the Weibull distribution under the generalized progressive hybrid censoring scheme, Reference [12] providing the maximum likelihood and Bayes estimators of the entropy for the Weibull distribution under a generalized progressive hybrid censoring scheme, Reference [13] studying the estimation of the entropy for the generalized exponential distribution based on record values, Reference [14] discussing the Shannon entropy for the Lomax distribution based on the generalized progressively hybrid censoring scheme and Reference [15] investigating point and interval estimation of the Shannon entropy for the inverse Weibull distribution under multiple censored data.

This paper provides a contribution to the estimation of the Rényi and *q*-entropies for the inverse Lomax distribution when multiple censored data are observed. Let us now motivate the consideration of the inverse Lomax distribution in this setting, as well as the multiple censored data. First and foremost, the inverse Lomax (IL) distribution is a lifetime distribution, defined as the distribution of the reciprocal of a random variable following the famous Lomax (L) distribution. Mathematically, the L distribution is defined by the cumulative distribution function (cdf) and pdf given as

$$\begin{array}{c} F_{*}\left(x;\alpha ,\theta \right)=1-{\left(1+\frac{x}{\theta }\right)}^{-\alpha },\;f_{*}\left(x;\alpha ,\theta \right)=\frac{\alpha }{\theta }{\left(1+\frac{x}{\theta }\right)}^{-\alpha -1},\;\;x,\alpha ,\theta >0, \end{array}$$

respectively. Here, α>0 is a shape parameter and θ>0 is a scale parameter. The essential of the L distribution can be found in References [16,17,18]. Thus, for a random variable *X* having the L distribution, the random variable Y=1/X follows the IL distribution with cdf and pdf given by

(3) $$\begin{array}{c} F\left(x;\alpha ,\theta \right)={\left(1+\frac{x^{-1}}{\theta }\right)}^{-\alpha },\;f\left(x;\alpha ,\theta \right)=\frac{\alpha }{\theta }x^{-2}{\left(1+\frac{x^{-1}}{\theta }\right)}^{-\alpha -1},\;\;x,\alpha ,\theta >0, \end{array}$$

respectively. Among the advantages of the IL distribution, the corresponding probability functions are tractable, it is parsimonious in parameters and possesses a non-monotonic hazard rate function; it possesses decreasing and upside-down bathtub shapes. The practical usefulness of the related model is illustrated in Reference [19] for its application to the analyses of geophysical data and in Reference [20] for its application in economics and actuarial sciences. Also, we refer to Reference [21] for the estimation of the reliability parameter via Type II censoring samples, in Reference [22] for the estimation of the parameters based on hybrid censored samples, and in Reference [23] for the Bayesian estimation of the two-component mixture of the IL distribution under the Type I censoring scheme. Also, recent studies have proposed extensions of the IL distributions for further purposes. In this regard, let us cite Reference [24] for the inverse power Lomax (or power IL) distribution, Reference [25] for the Weibull IL distribution, Reference [26] for the alpha power transformed IL distribution, Reference [27] for the Marshall-Olkin IL distribution and Reference [28] for the odd generalized exponentiated IL distribution.

However, as far as we know, despite its interest, the estimation of entropy measures for the IL distribution, such as the Rényi and *q*-entropies, remains an unexplored aspect. This study fills this gap by considering this problem under the realistic scenario of multiple censored data. This scenario commonly occurs where several censoring levels logically exist, which is often the case for many applications in life testing and survival analysis. We refer the reader to References [29,30,31], as well as the recent estimation studies of Reference [32] and Reference [15]. In our statistical framework, after investigating the maximum likelihood estimates of α and θ, estimates for the Rényi and *q*-entropies are derived. Then, two-sided approximate confidence intervals of the Rényi and *q*-entropies are discussed. A complete numerical study is performed, showing the favorable behavior of the obtained estimates at various sample sizes. In particular, the mean squared errors, approximate confidence intervals along with the corresponding average lengths are used as benchmarks. Our numerical investigations show that, when the sample size increases, the values of the mean squared errors and average lengths decrease. Also, when the censoring level decreases, the considered of Rényi and *q*-entropies estimates approach the true value. Two real life data sets, one physiological data set and one economic data set, are used to illustrate the findings.

The rest of the article is arranged as follows. The Rényi and *q*-entropies for the IL distribution are expressed in Section 2. Section 3 studies their estimation under multiple censored data. Simulation and numerical results are given in Section 4. An application to real data sets is presented in Section 5. The article ends with some concluding remarks in Section 6.

## 2. Expressions of the Rényi and *q*-Entropies

Let *X* be a random variable following the IL distribution with parameters α and θ. Then, by (1) and (3) with φ=(α,θ), the Rényi entropy of *X* is given by

$$\begin{array}{c} I_{\delta }\left(X;\alpha ,\theta \right)=\frac{1}{1-\delta }\log\left[\int_{0}^{+\infty }\frac{\alpha^{\delta }}{\theta^{\delta }}x^{-2\delta }{\left(1+\frac{x^{-1}}{\theta }\right)}^{-\delta (\alpha +1)}dx\right], \end{array}$$

where δ≠1 and δ>0. It follows from the standard equivalence results and criterion for Riemann integrability that $I_{\delta }\left(X;\alpha ,\theta \right)$ exists if and only if 2δ−1>0 and δ(α−1)+1>0. So the final conditions are δ≠1, δ>1/2 and δ(α−1)+1>0. Note that the last condition is alway satisfied if α≥1, which will be considered in the coming simulation study.

Under these conditions, by applying the change of variables $y=x^{-1}/\theta$, we can rewrite the integral term as

(4) $$\begin{array}{cc} \int_{0}^{+\infty }\frac{\alpha^{\delta }}{\theta^{\delta }}x^{-2\delta }{\left(1+\frac{x^{-1}}{\theta }\right)}^{-\delta (\alpha +1)}dx & =\alpha^{\delta }\theta^{\delta -1}\int_{0}^{+\infty }y^{2(\delta -1)}{\left(1+y\right)}^{-\delta (\alpha +1)}dy\\ \; & =\alpha^{\delta }\theta^{\delta -1}B\left(2\delta -1,\delta (\alpha -1)+1\right), \end{array}$$

where B(x,y) refers to the beta function defined by $B\left(x,y\right)=\int_{0}^{+\infty }t^{x-1}{\left(1+t\right)}^{-(x+y)}dt$, x,y>0 or, alternatively, $B\left(x,y\right)=\int_{0}^{1}t^{x-1}{\left(1-t\right)}^{y-1}dt$.

Therefore, after some algebraic manipulations, we get

(5) $$\begin{array}{cc} I_{\delta }\left(X;\alpha ,\theta \right) & =\frac{1}{1-\delta }\log\left[\alpha^{\delta }\theta^{\delta -1}B\left(2\delta -1,\delta (\alpha -1)+1\right)\right]\\ \; & =\frac{\delta }{1-\delta }\log\alpha -\log\theta +\frac{1}{1-\delta }\log\left[B\left(2\delta -1,\delta (\alpha -1)+1\right)\right], \end{array}$$

with δ≠1, δ>1/2 and δ(α−1)+1>0.

Similarly, based on (2) with φ=(α,θ) and (4) applied with δ=q, the *q*-entropy of *X* is given by

(6) $$\begin{array}{cc} H_{q}\left(X;\alpha ,\theta \right) & =\frac{1}{q-1}\left[1-\int_{0}^{+\infty }\frac{\alpha^{q}}{\theta^{q}}x^{-2q}{\left(1+\frac{x^{-1}}{\theta }\right)}^{-q(\alpha +1)}dx\right]\\ \; & =\frac{1}{q-1}\left[1-\alpha^{q}\theta^{q-1}B\left(2q-1,q(\alpha -1)+1\right)\right], \end{array}$$

with q≠1, q>1/2 and q(α−1)+1>0. For practical purposes, (5) and (6) are the required expressions of Rényi and *q*-entropies of *X*, written as simple functions of the parameters α and θ.

## 3. Estimation of Rényi and *q*-Entropies

### 3.1. Mathematical Basics on Multiple Censored Data Setting

For our estimation study, we consider the situation of multiple censored data (including the type I and type II censoring), following the setting of Reference [30] (Section 1.3.2). We may also refer to Reference [25] in the context of the inverse Weibull distribution. The general framework can be summarized as follows. Let *X* be a random variable having the cdf and pdf given by f(x;φ) and F(x;φ), respectively. Based on *n* units under a certain test, we get *n* values $x_{1},\ldots ,x_{n}$ of which

- $n_{f}$ values are (independent) observations of *X* for $n_{f}$ failed units,
- $n_{m}$ values are (independent) observations of *X* for $n_{m}$ censored (nonfailed) units,

with $n_{m}+n_{f}=n$. Then, the likelihood function for φ can be expressed as

$$\begin{array}{c} L\left(\varphi \right)=K\prod_{i=1}^{n}{\left[f(x_{i};\varphi )\right]}^{\varepsilon_{i,f}}{\left[1-F(x_{i};\varphi )\right]}^{\varepsilon_{i,m}}, \end{array}$$

where $\varepsilon_{i,f}=1$ if the *i*th unit failed, and 0 otherwise (so $\sum_{i=1}^{n}\varepsilon_{i,f}=n_{f}$), $\varepsilon_{i,m}=1$ if the *i*th unit censored, and 0 otherwise (so $\sum_{i=1}^{n}\varepsilon_{i,m}=n_{m}$), and *K* denotes a secondary constant (independent of φ). Then, the maximum likelihood estimates (MLEs) of φ are obtained by maximizing L(φ) with respect to φ. All the details in this regard can be found in Reference [30] (Section 1.3.2).

### 3.2. Considered Estimates

Hence, if a random variable *X* follows the IW distribution with parameters α and θ, based on (3), the likelihood function for φ=(α,θ) can be expressed as

$$\begin{array}{cc} L(\alpha ,\theta ) & =K\prod_{i=1}^{n}{\left[f(x_{i};\alpha ,\theta )\right]}^{\varepsilon_{i,f}}{\left[1-F(x_{i};\alpha ,\theta )\right]}^{\varepsilon_{i,m}}\\ \; & =K\prod_{i=1}^{n}{\left[\frac{\alpha }{\theta }x_{i}^{-2}{\left(1+\frac{x_{i}^{-1}}{\theta }\right)}^{-\alpha -1}\right]}^{\varepsilon_{i,f}}{\left[{1-\left(1+\frac{x_{i}^{-1}}{\theta }\right)}^{-\alpha }\right]}^{\varepsilon_{i,m}}. \end{array}$$

The MLEs are thus obtained by maximizing L(α,θ) with respect to α and θ. In this regard, the log-likelihood function is useful, and can be expressed as

$$\begin{array}{cc} \log[L(\alpha ,\theta )] & =\log K+n_{f}\log\alpha -n_{f}\log\theta -2\sum_{i=1}^{n}\varepsilon_{i,f}\log\left(x_{i}\right)-\left(\alpha +1\right)\sum_{i=1}^{n}\varepsilon_{i,f}\log\left(1+\frac{x_{i}^{-1}}{\theta }\right)\\ \; & +\sum_{i=1}^{n}\varepsilon_{i,m}\log\left[{1-\left(1+\frac{x_{i}^{-1}}{\theta }\right)}^{-\alpha }\right]. \end{array}$$

Then, the first partial derivatives of log[L(α,θ)] with respect to α and θ, are obtained as follows

$$\begin{array}{c} \frac{\partial \log[L(\alpha ,\theta )]}{\partial \alpha }=\frac{n_{f}}{\alpha }-\sum_{i=1}^{n}\varepsilon_{i,f}\log\left(1+\frac{x_{i}^{-1}}{\theta }\right)+\sum_{i=1}^{n}\frac{\varepsilon_{i,m}{\left(1+\frac{x_{i}^{-1}}{\theta }\right)}^{-\alpha }\log\left(1+\frac{x_{i}^{-1}}{\theta }\right)}{1-{\left(1+\frac{x_{i}^{-1}}{\theta }\right)}^{-\alpha }} \end{array}$$

and

$$\begin{array}{c} \frac{\partial \log[L(\alpha ,\theta )]}{\partial \theta }=-\frac{n_{f}}{\theta }+\left(\alpha +1\right)\sum_{i=1}^{n}\frac{\varepsilon_{i,f}}{\theta^{2}x_{i}+\theta }-\frac{\alpha }{\theta^{2}}\sum_{i=1}^{n}\frac{\varepsilon_{i,m}x_{i}^{-1}{\left(1+\frac{x_{i}^{-1}}{\theta }\right)}^{-\alpha -1}}{1-{\left(1+\frac{x_{i}^{-1}}{\theta }\right)}^{-\alpha }}. \end{array}$$

Hence, the MLEs of α and θ are determined by solving the following equations: ∂log[L(α,θ)]/∂α=0 and ∂log[L(α,θ)]/∂θ=0, simultaneously. These equations cannot be solved analytically, so numerical iterative techniques must be applied in this regard. For the purpose of this study, the MLEs of α and θ are denoted by $\hat{\alpha }$ and $\hat{\theta }$, respectively. Hence, based on (5) and (6), owing to the plugging approach, natural estimates for the entropies $I_{\delta }\left(X;\alpha ,\theta \right)$ and $H_{q}\left(X;\alpha ,\theta \right)$, are, respectively, given by

(7) $$\begin{array}{c} {\hat{I}}_{\delta }\left(X\right)={\hat{I}}_{\delta }\left(X;\hat{\alpha },\hat{\theta }\right)=\frac{\delta }{1-\delta }\log\hat{\alpha }-\log\hat{\theta }+\frac{1}{1-\delta }\log\left[B\left(2\delta -1,\delta (\hat{\alpha }-1)+1\right)\right], \end{array}$$

with δ≠1, δ>1/2 and $\delta (\hat{\alpha }-1)+1>0$, and

(8) $$\begin{array}{c} {\hat{H}}_{q}\left(X\right)={\hat{H}}_{q}\left(X;\hat{\alpha },\hat{\theta }\right)=\frac{1}{q-1}\left[1-{\hat{\alpha }}^{q}{\hat{\theta }}^{q-1}B\left(2q-1,q(\hat{\alpha }-1)+1\right)\right], \end{array}$$

with q≠1, q>1/2 and $q(\hat{\alpha }-1)+1>0$.

### 3.3. Confidence Intervals

Owing to the invariance property, ${\hat{I}}_{\delta }\left(X\right)$ and ${\hat{H}}_{q}\left(X\right)$ are also the MLEs of $I_{\delta }\left(X;\alpha ,\theta \right)$ and $H_{q}\left(X;\alpha ,\theta \right)$, respectively. Therefore, the well-known theory of the maximum likelihood method can be applied to ${\hat{I}}_{\delta }\left(X\right)$ and ${\hat{H}}_{q}\left(X\right)$. In particular, invoking the so-called Delta theorem, under some technical regularity conditions, the subjacent asymptotic distribution of ${\hat{I}}_{\delta }\left(X\right)$ can be approximated by the normal distribution with mean $I_{\delta }\left(X;\alpha ,\theta \right)$ and variance $DJ^{-1}D^{T}$, where $J^{-1}$ denotes the inverse of the observed information matrix and $D=\left(\partial I_{\delta }\left(X;\alpha ,\theta \right)/\partial \alpha ,\partial I_{\delta }\left(X;\alpha ,\theta \right)/\partial \theta \right){|}_{\alpha =\hat{\alpha },\theta =\hat{\theta }}$, both can be determined from (5). Therefore, the two-sided approximate confidence interval for the Rényi entropy at the confidence level 100(1−ν)% with ν∈(0,1) is given by $\Upsilon_{I_{\delta }}\left(\nu \right)=\left[L_{I_{\delta }}\left(\nu \right),U_{I_{\delta }}\left(\nu \right)\right]$ (so $P\left[I_{\delta }\left(X\right)\in \Upsilon_{I_{\delta }}\left(\nu \right)\right]=1-\nu$), where

$$\begin{array}{c} L_{I_{\delta }}\left(\nu \right)={\hat{I}}_{\delta }\left(X\right)-z_{\nu /2}{\hat{\sigma }}_{\left({\hat{I}}_{\delta }\left(X\right)\right)},\;U_{I_{\delta }}\left(\nu \right)={\hat{I}}_{\delta }\left(X\right)+z_{\nu /2}{\hat{\sigma }}_{\left({\hat{I}}_{\delta }\left(X\right)\right)}, \end{array}$$

${\hat{\sigma }}_{\left({\hat{I}}_{\delta }\left(X\right)\right)}={\left[DJ^{-1}D^{T}\right]}^{1/2}$ and $z_{\nu /2}$ is the 100(1−ν/2) standard normal percentile. A similar result holds for ${\hat{H}}_{q}\left(X\right)$. We also refer to References [15,31] for more detail.

## 4. Simulation Study

Here, a simulation study is assessed to investigate the performance of the Rényi and *q*-entropies estimates given by (7) and (8), respectively. In this regard, we use the mean squared errors (MSEs), two-sided approximate confidence intervals along with their corresponding average lengths (ALs) (i.e., defined AL is the average value of U-L, where L and U denotes the lower and upper bounds of the corresponding interval, respectively) based on multiple censored data (or samples). We adopt the methodology of Reference [15]. Thus, the following procedure is conducted:

- 3000 random samples of sizes n=50, 100, 150, 200 and 300 are generated from the IL distribution based on multiple censored sample.
- The values of parameters are selected as
  Set1: (α=1.2,θ=2), Set2: (α=1.5,θ=2).
  For the failures at censoring level (CL), we arbitrary chose CL =0.5 and 0.7 (for instance, CL =0.7 means that the observations are based on 30% failed units and 70% censored units).
- The true values for $I_{\delta }\left(X;\alpha ,\theta \right)$ and $H_{q}\left(X;\alpha ,\theta \right)$ given by (5) and (6), and the average estimates ${\hat{I}}_{\delta }\left(X\right)$ and ${\hat{H}}_{q}\left(X\right)$ given by (7) and (8) are calculated, respectively. Different values for δ and *q* are considered.
- Finally, the average of the obtained estimates, MSEs and ALs with level 95% (so ν=0.05) are computed.

All the numerical results are presented in Table 1, Table 2, Table 3 and Table 4 for the Rényi entropy, and Table 5, Table 6, Table 7 and Table 8 for the *q*-entropy. All is calculated by the use of the mathematical software Mathcad.

Figure 1, Figure 2, Figure 3 and Figure 4 provide a graphical approach of the MSEs and ALs of the Rényi entropy estimates, whereas Figure 5, Figure 6, Figure 7 and Figure 8 provide a graphical approach of the MSEs and ALs of the *q*-entropy estimates.

Here, some remarks can be formulated about the behavior of the Rényi and *q*-entropies estimates according to Table 1, Table 2, Table 3, Table 4, Table 5, Table 6, Table 7 and Table 8, and Figure 1, Figure 2, Figure 3, Figure 4, Figure 5, Figure 6, Figure 7 and Figure 8:

- The MSEs of ${\hat{I}}_{\delta }\left(X\right)$ decrease as the sample size increases.
- The ALs of ${\hat{I}}_{\delta }\left(X\right)$ decrease as the sample size increases.
- The MSEs of ${\hat{I}}_{\delta }\left(X\right)$ increase when the value of δ increases.
- The ALs of ${\hat{I}}_{\delta }\left(X\right)$ increase when the value of δ increases.
- The MSEs of ${\hat{H}}_{q}\left(X\right)$ decrease as the sample size increases.
- The ALs of ${\hat{H}}_{q}\left(X\right)$ decrease as the sample size increases.
- The MSEs of ${\hat{H}}_{q}\left(X\right)$ decrease when the value of *q* increases.
- The ALs of ${\hat{H}}_{q}\left(X\right)$ decrease when the value of *q* increases.
- In almost situations the MSE of ${\hat{I}}_{\delta }\left(X\right)$ at CL =0.5 is less than the MSE of ${\hat{I}}_{\delta }\left(X\right)$ at CL =0.7.
- In almost situations the MSE of ${\hat{H}}_{q}\left(X\right)$ at CL =0.5 is less than the MSE of ${\hat{H}}_{q}\left(X\right)$ at CL =0.7.

These facts prove the good accuracy of our entropy estimates, which are logically recommended for further practical purposes.

## 5. Application

In this Section, two real life data sets are used to illustrate the finding, both described below.

The first data set is a physiological data set extracted from Reference [33]. It concerns twenty Duchenne patients (6–18 years age) with classical type of the muscular dystrophy. The Electrocardiography of these 20 patients based on the heart rate is given below in Table 9.

The second data set is an economic data set extracted from the following electronic address: https://tradingeconomics.com/pakistan/consumer-price-index-cpi. It refers to Pakistan Consumer Price Index (CPI) in Pakistan from May 2019 to April 2020. The data are collected in Table 10.

Then, based on the data, adopting the multiple censored data scheme, we apply ${\hat{I}}_{\delta }\left(X\right)$ and ${\hat{H}}_{q}\left(X\right)$ to estimate $I_{\delta }\left(X;\alpha ,\theta \right)$ and $H_{q}\left(X;\alpha ,\theta \right)$ where *X* denotes the considered random variables of interest, assuming to follow the IL distribution. Different values for CL, δ and *q* are considered. The obtained numerical results are displayed in Table 11 and Table 12 for the first and second data sets, respectively.

Thus, Table 11 and Table 12 show some numerical values of estimated entropies in a concrete scenario, following the multiple censored data scheme. We see that the results depends on the entropy parameters (δ or *q*), and also, the value for CL, beyond the standard complete standard (which can be obtained by taking CL =0).

## 6. Concluding Remarks

This article studies the estimation of the Rényi and *q*-entropies for inverse Lomax distribution under multiple censored data. We propose an efficient estimation strategy by using the maximum likelihood and plugging methods. The behavior of the Rényi and *q*-entropies estimates are calculated in terms of their mean squared errors and average lengths (depending on two-sided approximate confidence intervals). Numerical results are provided, showing that, as the sample size increases, the mean squared errors of our estimates decrease. Also, it can be observed that, as the sample size increases, the average lengths of our estimates decreases. Thus, the proposed estimates reveal to be efficient, providing new useful tools with potential applicability in many applied situations dealing with entropy of the inverse Lomax distribution. The article ends by presenting an application to two real life data sets.

## Figures and Tables

**Figure 1:**
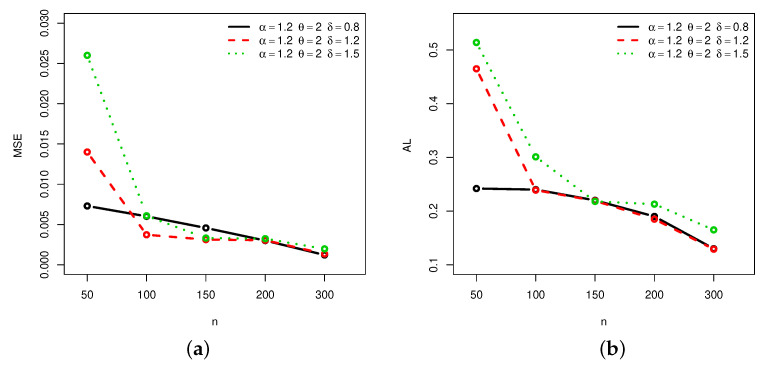
(**a**) Mean squared errors (MSEs) and (**b**) average lengths (ALs) of Rényi entropy estimates for different sample sizes at Set1 and CL =0.5.

**Figure 2:**
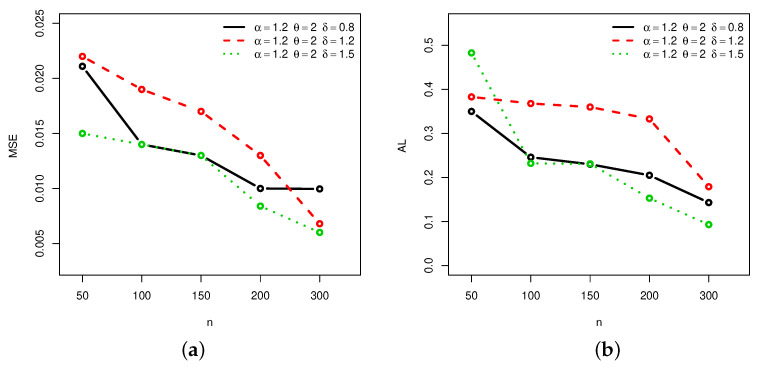
(**a**) MSEs and (**b**) ALs of Rényi entropy estimates for different sample sizes at Set1 and CL =0.7.

**Figure 3:**
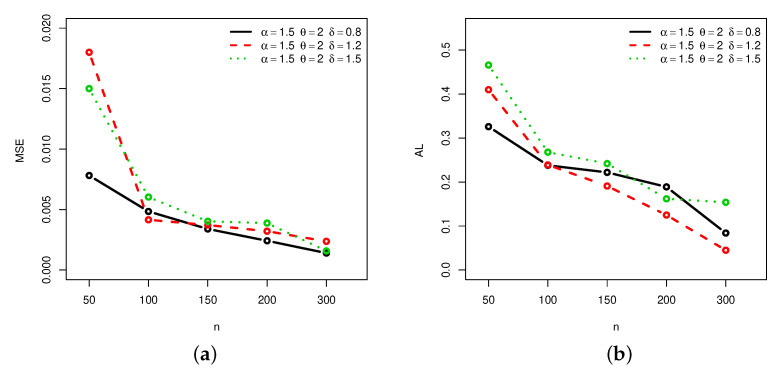
(**a**) MSEs and (**b**) ALs of Rényi entropy estimates for different sample sizes at Set2 and CL =0.5.

**Figure 4:**
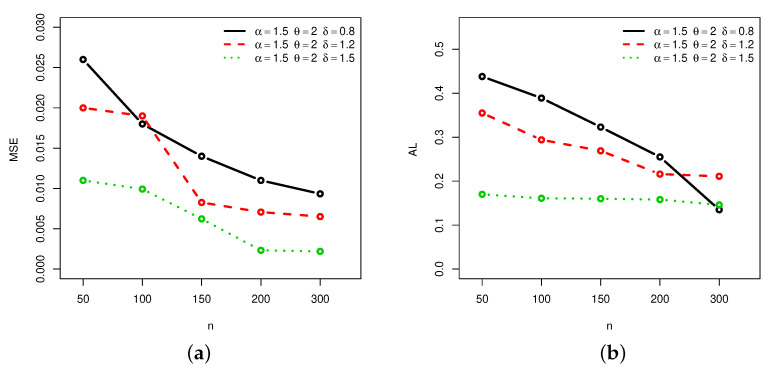
(**a**) MSEs and (**b**) ALs of Rényi entropy estimates for different sample sizes at Set2 and CL =0.7.

**Figure 5:**
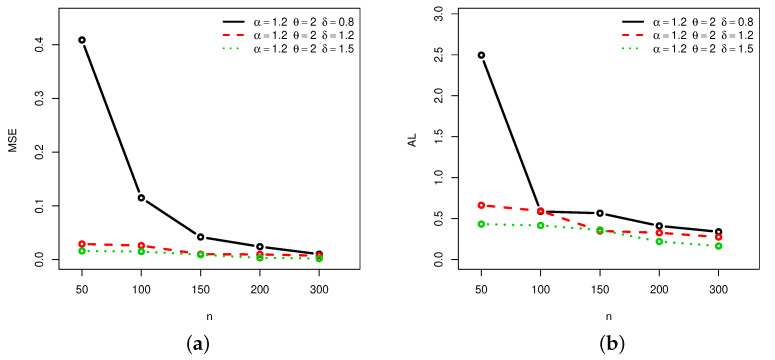
(**a**) MSEs and (**b**) ALs of *q*-entropy estimates for different sample sizes at Set1 and CL =0.5.

**Figure 6:**
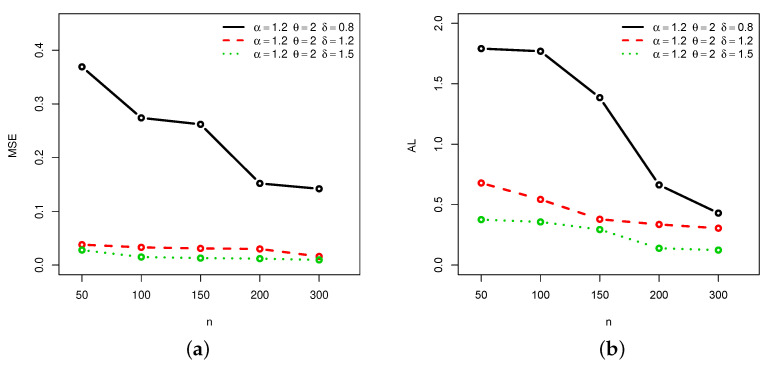
(**a**) MSEs and (**b**) ALs of *q*-entropy estimates for different sample sizes at Set1 and CL =0.7.

**Figure 7:**
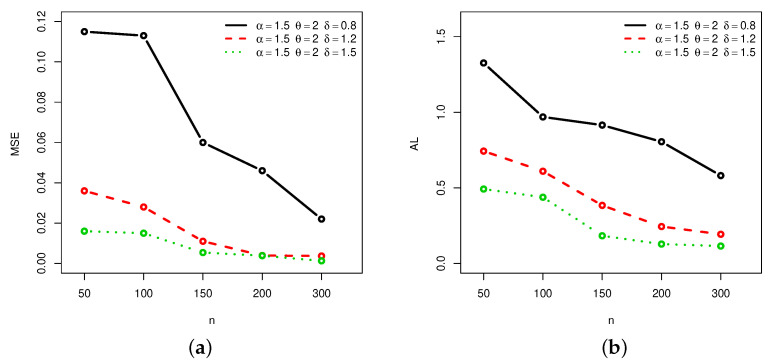
(**a**) MSEs and (**b**) ALs of *q*-entropy estimates for different sample sizes at Set2 and CL =0.5.

**Figure 8:**
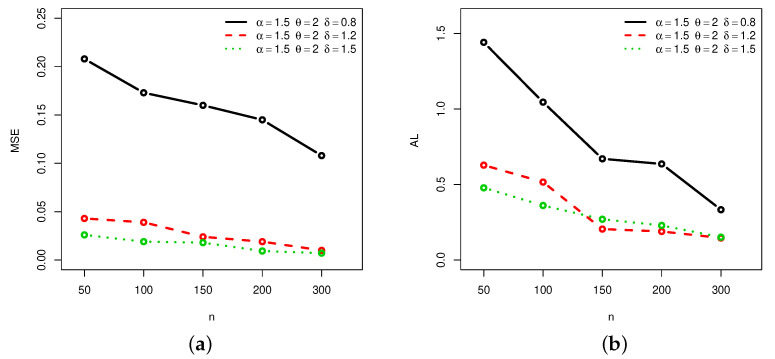
(**a**) MSEs and (**b**) ALs of *q*-entropy estimates for different sample sizes at Set2 and CL =0.7.

**Table 1:** Rényi entropy estimates at Set1 and censoring level (CL) =0.5.

*n*	δ=0.8				δ=1.2				δ=1.5			
	Exact Value	Estimates	MSE	AL	Exact Vlaue	Estimates	MSE	AL	Exact Value	Estimates	MSE	AL
50	0.9	0.841	7.301 *	0.242	0.534	0.547	0.014	0.465	0.413	0.319	0.026	0.514
100		0.853	6.014 *	0.24		0.533	3.727 *	0.239		0.426	6.08 *	0.301
150		0.937	4.572 *	0.223		0.534	3.123 *	0.219		0.398	3.316 *	0.218
200		0.875	3.006 *	0.19		0.563	3.052 *	0.185		0.431	3.242 *	0.212
300		0.896	1.217 *	0.136		0.55	1.348 *	0.129		0.427	1.974 *	0.165

* indicates that the value multiply $10^{-3}$.

**Table 2:** Rényi entropy estimates at Set1 and CL =0.7.

*n*	δ=0.8				δ=1.2				δ=1.5			
	Exact Value	Estimates	MSE	AL	Exact Value	Estimates	MSE	AL	Exact Value	Estimates	MSE	AL
50	0.9	1.015	0.021	0.351	0.534	0.644	0.022	0.383	0.413	0.511	0.015	0.483
100		1.003	0.014	0.246		0.618	0.019	0.368		0.506	0.014	0.232
150		1	0.013	0.23		0.616	0.017	0.36		0.501	0.013	0.231
200		0.964	0.01	0.205		0.612	0.013	0.333		0.483	8.4 *	0.153
300		0.993	9.959 *	0.143		0.603	6.797 *	0.179		0.463	6.007 *	0.093

* indicates that the value multiply $10^{-3}$.

**Table 3:** Rényi entropy estimates at Set2 and CL =0.5.

*n*	δ=0.8				δ=1.2				δ=1.5			
	Exact Value	Estimates	MSE	AL	Exact Value	Estimates	MSE	AL	Exact Value	Estimates	MSE	AL
50	1.008	1.038	7.815 *	0.326	0.652	0.739	0.018	0.41	0.535	0.555	0.015	0.466
100		1.026	4.843 *	0.238		0.618	4.152 *	0.239		0.551	6.032 *	0.268
150		0.994	3.394 *	0.222		0.672	3.718 *	0.191		0.55	4.025 *	0.242
200		0.999	2.418 *	0.189		0.649	0.321 *	0.125		0.542	3.882 *	0.162
300		1.009	1.401 *	0.084		0.65	0.237 *	0.045		0.53	1.578 *	0.154

* indicates that the value multiply $10^{-3}$.

**Table 4:** Rényi entropy estimates at Set2 and CL =0.7.

*n*	δ=0.8				δ=1.2				δ=1.5			
	Exact Value	Estimates	MSE	AL	Exact Value	Estimates	MSE	AL	Exact Value	Estimates	MSE	AL
50	1.008	1.159	0.026	0.438	0.652	0.799	0.02	0.355	0.535	0.635	0.011	0.17
100		1.099	0.018	0.389		0.765	0.019	0.294		0.626	9.926 *	0.161
150		1.059	0.014	0.323		0.732	8.262 *	0.269		0.595	6.224 *	0.16
200		1.036	0.011	0.255		0.715	7.068 *	0.216		0.575	2.324 *	0.158
300		1.01	9.338 *	0.135		0.712	6.514 *	0.211		0.564	2.189 *	0.146

* indicates that the value multiply $10^{-3}$.

**Table 5:** *q*-entropy estimates at Set1 and CL =0.5.

*n*	q=0.8				q=1.2				q=1.5			
	Exact Value	Estimates	MSE	AL	Exact Value	Estimates	MSE	AL	Exact Value	Estimates	MSE	AL
50	8.569	8.876	0.409	2.496	−2.91	−2.839	0.029	0.663	−0.243	−0.18	0.016	0.433
100		8.393	0.115	0.587		−2.858	0.026	0.594		−0.221	0.015	0.416
150		8.508	0.042	0.566		−2.95	9.99 *	0.349		−0.227	9.035 *	0.362
200		8.517	0.024	0.411		−2.925	9.573 *	0.327		−0.233	3.265 *	0.22
300		8.526	0.01	0.339		−2.914	7.183 *	0.275		−0.251	1.895 *	0.165

* indicates that the value multiply $10^{-3}$.

**Table 6:** *q*-entropy estimates at Set1 and CL =0.7.

*n*	q=0.8				q=1.2				q=1.5			
	Exact Value	Estimates	MSE	AL	Exact Value	Estimates	MSE	AL	Exact Value	Estimates	MSE	AL
50	8.569	8.969	0.369	1.791	−2.91	−2.752	0.038	0.679	−0.243	−0.081	0.028	0.376
100		8.943	0.274	1.769		−2.762	0.033	0.543		−0.144	0.015	0.357
150		8.939	0.262	1.385		−2.774	0.031	0.379		−0.176	0.013	0.294
200		8.905	0.152	0.663		−2.814	0.03	0.336		−0.181	0.012	0.139
300		8.834	0.142	0.43		−2.887	0.016	0.305		−0.223	9.603 *	0.124

* indicates that the value multiply $10^{-3}$.

**Table 7:** *q*-entropy estimates at Set2 and CL =0.5.

*n*	q=0.8				q=1.2				q=1.5			
	Exact Value	Estimates	MSE	AL	Exact Value	Estimates	MSE	AL	Exact Value	Estimates	MSE	AL
50	8.955	8.628	0.115	1.326	−2.703	−2.584	0.036	0.743	−0.08	−0.024	0.016	0.492
100		8.727	0.113	0.969		−2.641	0.028	0.61		−0.027	0.015	0.438
150		9.032	0.06	0.915		−2.733	0.011	0.384		−0.126	5.395 *	0.183
200		8.981	0.046	0.805		−2.711	3.923 *	0.244		−0.101	3.845 *	0.128
300		8.972	0.022	0.581		−2.698	3.703 *	0.193		−0.1	1.28 *	0.115

* indicates that the value multiply $10^{-3}$.

**Table 8:** *q*-entropy estimates at Set2 and CL =0.7.

*n*	q=0.8				q=1.2				q=1.5			
	Exact Value	Estimates	MSE	AL	Exact Vaule	Estimates	MSE	AL	Exact Value	Estimates	MSE	AL
50	8.955	9.378	0.208	1.441	−2.703	−2.514	0.043	0.628	−0.08	0.08	0.026	0.478
100		9.309	0.173	1.045		−2.555	0.039	0.516		0.046	0.019	0.361
150		9.292	0.16	0.67		−2.557	0.024	0.205		0.036	0.018	0.27
200		9.152	0.145	0.636		−2.592	0.019	0.189		−4.67 *	9.329 *	0.229
300		9.147	0.108	0.333		−2.649	0.01	0.146		−0.05	7.114 *	0.151

* indicates that the value multiply $10^{-3}$.

**Table 9:** First data set: Heart rate data for twenty Duchenne patients.

80	90	90	94	100	90	103	100	116	102
112	140	120	120	100	100	120	80	120	100

**Table 10:** Second data set: Consumer Price Index (CPI) in Pakistan from May 2019 to April 2020.

245.94	246.82	252.46	255.94	257.87	260.46
263.59	262.82	266.97	266.245	266.87	267.12

**Table 11:** Estimated of Rényi and *q*-entropies at CL =0.5 and CL =0.7 for the first data set.

CL = 0.5				CL = 0.7			
**Rényi Entropy**		***q*** **-Entropy**		**Rényi Entropy**		***q*** **-Entropy**	
δ=0.8	δ=1.2	q=0.8	q=1.2	δ=0.8	δ=1.2	q=0.8	q=1.2
−4.768	−6.806	1.017	1.002	−4.594	−9.278	1.06	1.061

**Table 12:** Estimated of Rényi and *q*-entropies at CL = 0.5 and CL = 0.7 for the second data set.

CL = 0.5				CL = 0.7			
**Rényi Entropy**		***q*** **-Entropy**		**Rényi Entropy**		***q*** **-Entropy**	
δ=0.8	δ=1.2	q=0.8	q=1.2	δ=0.8	δ=1.2	q=0.8	q=1.2
−2.868	−3.746	1.024	1.004	−4.063	−4.411	1.033	1.015
